# Percutaneous sperm retrieval technique: A reliable and effective sperm retrieval procedure for ICSI in patients with obstructive azoospermia

**DOI:** 10.5935/1518-0557.20240092

**Published:** 2025

**Authors:** Thiago P. Furtado, Andrea Kauffman Zeh, Marcelo H. Furtado

**Affiliations:** 1 University of California, Los Angeles, USA; 2 MF Fertilidade Masculina, Belo Horizonte, Brazil; 3 Universidade Federal de Minas Gerais, Belo Horizonte, Brazil

**Keywords:** Obstructive azoospermia (OA), percutaneous sperm retrieval, intracytoplasmic sperm injection (ICSI), epididymal sperm, male infertility

## Abstract

**Objective:**

To study the reliability and effectiveness of the percutaneous sperm retrieval technique.

**Methods:**

We retrospectively analyzed the records of 123 consecutive patients with obstructive azoospermia who underwent percutaneous sperm retrieval and intracytoplasmic sperm injection (ICSI) using Percutaneous Epididymal Sperm Aspiration (PESA) with or without a rescue Testicular Sperm Aspiration (TESA). We compared patients who had the first sperm retrieval ever and patients who had more than one sperm retrieval performed. In addition, the rate of adequate sperm retrieved for ICSI and reproductive outcomes between the groups and literature were compared. The primary outcome was the rate of adequate sperm retrieved for ICSI; the secondary outcomes were reproductive.

**Results:**

The successful sperm retrieval was 157 in 157 attempts, with a cumulative sperm retrieval rate of 100%, whether the first or repeated retrieval attempt. The clinical pregnancy rate in patients who had the first sperm retrieval ever and patients who had more than one sperm retrieval performed was 36/108 (33.3%) *vs*. 15/29 (51.7%) (Pearson chi2(1) = 3.3088; *p* = 0.085), respectively. Other reproductive outcomes (fertilization rate, cleavage rate, and biochemical pregnancy) were also similar in both groups.

**Conclusions:**

PESA associated with or without rescue TESA is a reliable and effective strategy either for retrieving sperm for ICSI, demystifying the concerns about the reliability of repeated PESA, or for reproductive results. This information is reassuring, especially but not exclusively to places with limited resources.

## INTRODUCTION

Approximately one-third of the couples seeking infertility treatment have male factors as their primary indication. Azoospermia, defined as the complete absence of sperm in the ejaculate, responds to the infertility of 1% of all men and 10% to 15% of all infertile couples (Jarow *et al*., 1989). Men with obstructive azoospermia (OA) have normal spermatogenesis but have a mechanical blockage between the epididymis and the ejaculatory ducts or a totally or partially absent epididymis and vas deferens. OA represents 40% of all azoospermic men (Jarow *et al*., 1989) and occurs in up to 13.6% (Jequier, 1985) of patients presenting for fertility evaluation. As OA is characterized by the diffuse distribution of normal or nearnormal sperm production throughout the testes, in most cases, motile sperm may be found in the proximal portions of the epididymis and the testes (Jow *et al*., 1993).

Given the wide distribution of sperm throughout the testes and epididymis in men with OA, sperm retrieval is often possible using percutaneous or surgical techniques. However, each sperm retrieval procedure has different anesthetic requirements, safety profiles, required surgical skills, and sperm yields. For men with OA, Microsurgical Epidydimal Sperm Aspiration (MESA) is the gold standard for retrieving adequate motile sperm. However, Percutaneous Epididymal Sperm Aspiration (PESA) is a more cost-effective, minimally invasive technique that recovers sperm directly from the epididymis. PESA avoids the need for surgical microscopes, trained microsurgeons, and expensive hospital costs required by MESA. However, PESA may cause extensive scarring of the epididymis, making future epididymal sperm retrieval attempts more challenging (Cha *et al*., 1997; Jarow *et al*., 2010; Rosenlund *et al*., 1998; Silber *et al*., 1994). Also, it may be more challenging to find the more proximal and less senescent sperm with PESA than with MESA. With both PESA and MESA, if only senescent sperm from the distal epididymis were used, intracytoplasmic sperm injection (ICSI) results are not good (Silber *et al*., 1994; Tournaye *et al*., 1994). Moreover, the spermatozoa in the epididymal fluid retrieved by PESA are unsuitable for ICSI in up to 25% of men with OA. For these patients, it is always possible to find spermatozoa in the testis through Testicular Sperm Aspiration (TESA) or Testicular Sperm Extraction (TESE). Several works suggest that the combination of percutaneous epididymal and testicular sperm recovery procedures in patients with OA yields reasonable spermatozoa recovery rates, ranging between 51% and 100% (Craft *et al*., 1995a; Esteves *et al*., 2013; Glina *et al*., 2003; Hao *et al*., 2017; Kovac *et al*., 2014; Wood *et al*., 2002; Yafi & Zini, 2013). However, few studies have examined the reproductive outcomes following ICSI using sperm retrieved after repeated PESA or combined with other percutaneous epididymal sperm recovery procedures.

Herein, we retrospectively evaluated sperm recovery rates and reproductive outcomes of 123 consecutive men with OA who underwent one or more PESA as the primary treatment strategy and TESA when PESA did not result in adequate motile sperm for use in ICSI. We also reviewed the literature considering the reproductive outcomes of operative sperm retrieval in OA that will be of great interest to clinicians and scientists involved in the reproductive treatment of these patients, especially those without male fertility training.

## MATERIALS AND METHODS

### Patients

We retrospectively analyzed the records of 123 consecutive patients with OA who underwent percutaneous sperm retrieval and ICSI from January 2013 to September 2018. We excluded patients using anabolic steroids and with any risk factors for male infertility other than excurrent duct obstruction or functional obstruction. Azoospermia was confirmed following the examination of at least two centrifuged ejaculates, according to WHO guidelines (World Health Organization, 2021). In addition, the obstruction etiology (CBAVD, vasectomy, or infection) and previous PESAs were recorded.

All patients underwent a complete evaluation, including medical history, physical examination, and hormone profiling to check serum FSH, LH, and total testosterone. We also recorded the female and male ages at the time of ICSI.

This study was approved by the institutional committee on human research under the number CAAE 78267024.5.000.5128, ensuring that it conformed to the ethical guidelines of the 1975 Declaration of Helsinki, which was reviewed in 2013.

### Sperm retrieval procedures

All sperm retrieval procedures were performed by a single surgeon (M.H.F.). All procedures were performed at the same time as oocyte retrieval from the female partner and used immediately for ICSI. PESA was our primary treatment strategy in men with OA; however, TESA was performed in those men who did not produce adequate motile sperm with PESA alone.

### PESA

In brief, the patient’s genital region was washed with Chlorhexidine 2%. After local anesthesia of the cord with Lidocaine 1%, the epididymis was pinched between the thumb and forefinger. Aspiration of the epididymis was done using a 1mL syringe attached to an insulin needle with multiple passes without removing the needle from the initial insertion site. An embryologist placed the aspirate in a petri dish and examined it for motile epididymal spermatozoa under the inverted microscope (Nikon Eclipse Ti-S). The procedure was ended if the retrieved sperm was considered adequate for ICSI. If not, another aspiration was made on the same or the contralateral side. Whenever the search for adequate sperm on both epididymis failed, the surgeon proceeded with TESA.

### TESA

A 16F intravenous catheter was passed through the scrotal skin into the testicular tissue. The catheter was usually inserted into the anterolateral portion of the superior testicular pole at an oblique angle toward the medium and lower poles. After insertion, the catheter was coupled to an intravenous line cord extension and a 20 ml syringe mounted on a CAMECO syringe pistol. The testicular parenchyma was aspirated by creating a negative pressure and moving the angiocatheter forth and backward in different directions into the testicular tissue. If adequate spermatozoa for ICSI were found in the specimen obtained, they were used immediately. If not, the same procedure was performed on the contralateral side.

### ICSI

The ICSI procedures were all performed using an ovary stimulation protocol devised by the Reproductive Endocrinology and Infertility specialist. The most motile and best morphologically sperm were injected in each MII oocyte retrieved. On day 1, at 16-18 h after injection, all embryos were checked for fertilization. On day 3, all embryos were checked and scored according to the SART system (Racowsky *et al*., 2010), followed by transferring the best embryos to the uterus.

### Data collection and study groups

We determined the sperm retrieval success, oocyte fertilization and cleavage, and biochemical and clinical pregnancy. Retrieval was considered successful when an adequate number of motile sperm cells were recovered to proceed with ICSI. The present study analyzed two groups of patients: patients who underwent the first PESA ever for their first ICSI cycle (PESA group) and patients who underwent >1 PESA for repeated cycles of ICSI (PESA+ group). Failure of previous cycles was the reason for repeated cycles of ICSI.

We used the following definitions: (i) Fertilization rate as the total number of fertilized oocytes by the total number of mature oocytes retrieved; (ii) Cleavage rate as the total number of day-3 embryos by the total number of fertilized oocytes; (iii) Biochemical pregnancy, the rise of serum beta-human chorionic gonadotropin (beta-hCG) concentration ten days after ET and again two days later to confirm biochemical pregnancy; (iv) Clinical pregnancy, the visualization of a gestational sac by ultrasonography on the seventh week of gestation; and (v) Clinical pregnancy rate as the ratio between the number of clinical pregnancies and the number of initiated ICSI cycles.

### Statistical analysis

Baseline characteristics are presented using descriptive statistics: mean and standard deviation (SD) for normally distributed continuous data, median and interquartile range (IQR) for non-normally distributed continuous data, and proportions and frequencies for categorical data.

The statistical analysis compared the PESA group and PESA + group on mean male age, mean female partner age, number of successful adequate sperm retrieval for ICSI, and reproductive outcomes (fertilization rate, cleavage rate, number of biochemical pregnancies, and number of clinical pregnancy). Continuous variables were compared using the unpaired Student-t test if parametric assumptions were met; otherwise, the Wilcoxon rank sum test was used. Categorical outcomes comparisons were conducted by constructing 2x2 tables and using the Chisquare or Fisher’s exact test. We will use 2-sided *p*-values with alpha <0.05 significance level for all tests.

## RESULTS

There was no difference regarding mean male age, mean female partner age, rate of adequate sperm retrieved for ICSI, and reproductive outcomes between the PESA group and PESA+ group, whether accompanied or not by TESA (Table 1).

**Table 1:** Comparison between PESA and PESA+ groups.

	PESA	PESA+	p-value
Number of patients	123	25	-
Number of PESA	125	32	-
Male Age Years (mean, sd)	46.8 (8.3)	46.6 (8.8)	-
Female Age Years (mean, sd)	34.7 (5.5)	36.3 (5.1)	-
Fertilization Rate % (median, IQR)	75 (60, 90)	71.4 (50, 84.6)	-
Cleavage Rate % (median, IQR)	100 (90.9, 100)	100 (100, 100)	-
Biochemical Pregnancy n (%)	40/108 (37)	17/29 (58.6)	0.055^*^
Clinical Pregnancy n (%)	36/108 (33.3)	15/29 (51.7)	0.085^*^

* Fisher Exact test.

All 123 patients studied herein underwent at least one PESA. Most patients (*n*=98, 79.7%) underwent only one PESA in their lifetime, while 25 (20.3%) underwent two or more PESAs in the same epididymis (Table 1). Four patients performed up to three PESAs, one performed up to four PESAs, and one underwent five PESAs in the same testicle (Table 2). Thus, a total of 157 PESAs were considered in this analysis. The primary etiology of azoospermia was vasectomy (n=99; 83.2%) followed by idiopathic epidydimal obstruction (n=10; 8.4%), CBAVD (n=7; 5.9%), aspermia (n=3; 2.5%) including two patients with Spinal Cord Injury. In four patients, the primary etiology was unknown.

**Table 2:** Adequate spermatozoa retrieval rate for ICSI according to the number of PESA and the procedure performed.

Procedure			1^st^ PESA (n=125)		2^nd^ PESA (n=26)		3^rd^ PESA (n=4)		4^th^ PESA (n=1)		5^th^ PESA (n=1)
PESA n (cum. %)			114 (91.2%)		21 (80.8%)		4 (100%)		1 (100%)		0
Contralateral PESA n (cum.%)			5 (95.2%)		2 (88.5%)		-		-		0
TESA n (cum. %)			6 (100%)		3 (100%)		-		-		1 (100%)

The sperm retrieval yield in adequate spermatozoa in 140 of all 157 PESAs performed in the study (89.2%), whereas, in 17 (10.8%) of the PESAs, spermatozoa considered inadequate to do the ICSI procedure by the embryologist or absent could only be successfully retrieved following further retrieval procedures such as 7 of 12 (58.3%) contralateral PESA or 10 of 10 (100%) TESA. Adequate sperms to do the ICSI were retrieved in all cases, regardless of the number of PESAs performed in the same testicle or the need for further rescue procedures such as contralateral PESA or TESA (Table 2). There were only minor adverse effects like mild pain and discrete hematoma on the scrotum following PESA or TESA.

We examined our records for the reproductive outcomes of the ICSI cycles performed with sperm retrieved from our OA patients with PESA in combination with TESA. In our hands, no significant differences were found regarding the average fertilization and cleavage rates as well as the rates of biochemical and clinical pregnancies, independently of whether sperm were retrieved with a single or repeated PESAs in one same epididymis or by using further TESA to improve sperm recovery rate in the same testicles (Table 1).

## DISCUSSION

We retrospectively evaluated the records of 123 consecutive patients with OA who underwent 157 percutaneous sperm retrievals and ICSI to determine if these procedures were reliable and effective regarding successful sperm retrieval rates and reproductive outcomes being the first or repeated procedures. Our results showed that PESA is reliable for retrieving sperm for ICSI.

In all cases, we could retrieve sperms that are adequate for ICSI. Table 3 shows the studies reporting sperm retrieval rates using PESA as the first choice, followed by rescue with contralateral PESA, TESA, or TESE. Sperm retrieval with PESA alone produces excellent sperm recovery rates since most studies (10 in 15) found motile sperm in more than 80% of the cases studied. Furthermore, when epididymal and testicular retrievals were combined, the recovery performance was close to 100% in 9 of 15 studies. While Levine *et al*. (2003) had impressive results with a 91/94 (97%) success rate of sperm retrieval performing PESA, others, like Datta *et al*. (2016), Lin *et al*. (2000), and Dohle *et al*. (1998), presented low recoveries. Although Datta *et al*. (2016) showed a low sperm retrieval rate of 18/39 (46.1%) when PESA was used as the primary technique, the overall recovery rate increased to 34/44 (77.3%), combining PESA and TESA. Lin *et al*. (2000) recovered sperm from 66 of the 109 executed attempts (61%). When PESA failed, MESA was performed, and if both techniques failed, TESA was employed to provide motile sperm. In the end, all 109 attempts produced spermatozoa suitable for ICSI. The authors did not explain why they did not obtain sperm using PESA but did using MESA. The low rates of sperm recovery using PESA reported by Dohle *et al*. (1998) were probably due to the etiology of OA. They included 11 cases of failed microsurgical reconstruction, nine of CBAVD, and four of genital infection. Nevertheless, in the latter two studies, Lin *et al*. (2000) and Dohle *et al*. (1998), managed 100% sperm recovery by adding further testicular retrieval procedures.

**Table 3:** Literature survey on sperm retrieval successes of PESA as the first choice combined or not with TESA for later salvage performed in men with obstructive azoospermia.

Study (Year)	Patients n	Procedures n	Adequate Sperm Rate with PESA only n (%)	Rescue TESA Rate n (%)	Cumulative PESA + TESA Rate n (%)
This study	123	157	147/157 (93.6)	10/10 (100)	157/157 (100)
Craft *et al*. (1995a)	20	20	16/20 (80)	1/3 (33.3)	17/20 (85)
Craft *et al*. (1995b)	38	42	34/38 (89)	-	89 (34/38)
Datta *et al*. (2016)	44	39	18/39 (46.1)	34/44 (77.3)	-
Dohle *et al*. (1998)	-	29	18/29 (62)	9/11 (81.8)	27/29 (93)
Esteves *et al*. (2013)	146	146	114/146 (78.1)	28/32 (87.5)	142/146 (97)
Glina *et al*. (2003)	58	79	65/79 (82)	14/14 (100)	79/79 (100)
Kovac *et al*. (2014)	68	68	51/68 (75)	-	51/68 (75)
Levine *et al*. (2003)	94	94	91/94 (97)	3/3 (100)	94/94 (100)
Lin *et al*. (2000)	100	109	66/109 (61)	43/43 (100)	109/109 (100)
Meniru *et al*. (1997)	122	122	112/122 (91.8)	9/10 (90)	121/122 (99)
Rosenlund *et al*. (1998)	27	58	52/58 (89.7)	6/6 (100)	58/58 (100)
Tsirigotis *et al*. (1995)	36	40	32/40 (80)	8/8 (100)	40/40 (100)
Verza & Esteves (2008)	39	39	31/39 (81.5)	8/8 (100)	39/39 (100)
Yafi & Zini (2013)	255	255	216/255 (84.7)	48/48 (100)	255/255 (100)

Despite the reported sperm retrieval accomplishments, the blindness of the PESA procedure has led to fears of injuries to the epididymis, making it more challenging to find motile sperm for ICSI when repeated (Cha *et al*., 1997; Jarow *et al*., 2010; Rosenlund *et al*., 1998; Silber *et al*., 1994). In the present study, we analyzed the effect of repeated PESAs on sperm retrieval and found that PESA is repeatable. In the study, 26 of 32 patients of the PESA+ group (81.3%) had successful sperm retrieval by PESA only, and 32 of 32 (100%) after association with contralateral PESA and TESA in case the first side attempt failed. Besides the present study, only three other works analyzed the effect of repeated PESAs on sperm retrieval (Table 4). Glina *et al*. (2003) and Rosenlund *et al*. (1998) successfully recovered motile sperm in all cases that needed repeated PESA in the same epididymis (up to four times). Pasqualotto *et al*. (2003) also suggested that PESA can be repeated in the same epididymis; however, it presents a lower retrieval rate than the latter two studies and ours. While we do not have hard data on whether or not damages were inflicted on our patients’ epididymal tubules or testis, repeated PESA, with or without additional TESA, retrieved motile sperm in virtually all patients with encouraging reproductive outcomes.

**Table 4:** Literature survey on sperm retrieval successes according to the number of PESA combined with TESA performed in men with obstructive azoospermia.

Study (Year)	n	PESA n	TESA n	Overall Adequate sperm retrieval rate for ICSI n (%)	Overall TESA Rescue n (%)	1^st^ PESA n (%)	Rescue with TESA n (%)	2^nd^ PESA n (%)	Rescue with TESA n (%)	3^rd^ PESA n (%)	Rescue with TESA n (%)	4^th^ PESA n (%)
Glina *et al.* (2003)	58	79	14	65/79 (82)	14/14 (100)	47/58 (81)	11/11(100)	13/15 (87)	2/2 (100)	4/5 (80)	1/1 (100)	11/1 (100)
Rosenlund *et al.* (1998)	27	58	6	52/58 (90)	6/6 (100)	20/22 (91)	2/2 (100)	24/27 (89)	3/3 (100)	6/7 (86)	1/1 (100)	100 (2/2)
Pasqualotto *et al.* (2003)	19	22	**-**	-	-	-	-	8/22 (37.5)	-	-	-	-

Some interesting features can be highlighted from the literature list compiled in Table 5, particularly concerning our results. The fertilization rates obtained herein are higher than those reported by 5 of the 10 PESA sperm retrieval studies (Craft *et al*., 1995b; Levine *et al*., 2003; Meniru *et al*., 1997; Tsirigotis *et al*., 1996; Tsirigotis *et al*., 1995) and close to the remaining five PESA sperm retrieval publications. Of the 10 PESA sperm retrieval publications, only three indicated cleavage rates. The average cleavage rate of our cases was close to that of two studies (Tsirigotis *et al*., 1995; 1996). However, Meniru *et al*. (1997) found a lower average cleavage rate (73%). Only two of the 10 PESA sperm retrieval publications evaluated biochemical pregnancy (Craft *et al*., 1995b; Dozortsev *et al*., 2006), with an average rate of 66/170 (38.2%), close to the rate we found in our casuistry. Since most PESA sperm retrieval publications reported average clinical pregnancy rates ranging from 21.1% to 46% positive results per cycle, we conclude that our findings are within this range.

**Table 5:** Literature survey on reproductive results after sperm retrieval by PESA ± TESA.

Study (Year)	Patients n	Fertilization Rate %	Cleavage Rate %	Biochemical Pregnancy Rate n (%)	Clinical Pregnancy Rate n (%)
**This study**	**123**	**72.9**	**91.6**	**57/157 (36.3)**	**51/157 (32.5)**
Craft *et al*. (1995b)	38	33	-	10/38 (26.3)	8/38 (21.1)
Dozortsev *et al*. (2006)	-	77	-	56/132 (42.4)	49/132 (37)
Esteves *et al*. (2013)	-	-	-	-	67/146 (46)
Glina *et al*. (2003)	58	66.4	-	-	30/79 (38)
Kovac *et al*. (2014)	51	76	-	-	23/51 (45)
Levine *et al*. (2003)	-	58	-	-	31/78 (39)
Meniru *et al*. (1997)	122	54	73	-	38/122 (31)
Naru *et al*. (2008)	53	-	-	-	30/69 (43.5)
Pasqualotto *et al*. (2002)	56	-	-	-	24/68 (35)
Tsirigotis *et al*. (1995)	28	48	81	-	8/32 (25)
Tsirigotis *et al*. (1996)	-	53	85	-	18/59 (30.5)

Only Friedler *et al*. (1998) and Lin *et al*. (2000) compared sperm retrieved by PESA and MESA regarding some reproductive outcomes. Friedler *et al*. (1998) examined 17 PESA and seven MESA procedures and found similar rates of fertilization (56% and 55%, respectively), cleavage (88% and 94%, respectively), and clinical pregnancy per cycle (29.4% and 28.6%, respectively). Lin *et al*. (2000) examined 66 PESA and 40 MESA procedures. They found that the retrieved sperm by both techniques produced similar average fertilization rates (56% and 47%, respectively) and clinical pregnancy per cycle (39% and 45%, respectively).

Although the data reported herein were collected from a single clinic and a single surgeon, thus reducing the heterogeneity in the procedures performed, the present study has limitations. The sample size is relatively small, although it is one of the largest. Due to the retrospective nature of this study, our patient population may have had some inherent selection bias since, as expected, the leading cause of azoospermia in our series was vasectomy. However, all the other main causes of obstruction were represented in the present study. Furthermore, although they are not new and come from a single clinic and a single surgeon, which could be seen as a limitation due to idiosyncrasies in patient selection and surgical performance, the results of this study improve the generalizability of the procedure, which is essential for a procedure that is expected to be incorporated into health or social policy.

## CONCLUSION

In this study, we confirm that using a combination of percutaneous sperm retrieval techniques is a reliable strategy for obtaining sperm for ICSI from men with OA. Testicular recovery rescued the failed epididymal sperm retrievals. In our hands, repeated PESA procedures with or without additional TESA did not significantly affect reproductive outcomes, including fertilization, cleavage, and biochemical and clinical pregnancy rates.

Our results and the present review add to the expanding knowledge regarding sperm retrieval strategies. They may help infertility physicians to better counsel patients with OA and reassure non-trained physicians working in fertility clinics on how to proceed with sperm recovery, taking into consideration sperm retrieval performance and ICSI outcomes of each technique alone or in combination.
